# Our Advertisers

**Published:** 1875-08

**Authors:** 


*Our Advertisers.*

It is as much the duty of a medical journalist to direct his readers to good houses, selling pure drugs, as it is to indicate the use of the drugs themselves. Believing this as firmly as we do, our readers may be sure that any house advertised in our columns is worthy of confidence.

*Medical Colleges.*—The University of Pennsylvania and the Bellevue Hospital Medical Colleges.

These excellent Schools are rapidly building a name for themselves second to none in this country. Their Chairs are well filled, and their facilities for clinical instruction are unsurpassed by any Colleges in the United States. We cordially recommend these institutions to our readers and their students, as they have not adopted the pernicious system of free courses, that is rapidly flooding our country with men who are totally unfit to fill the position their diploma *seems* to entitle them to.

*Our Local Druggists.*—We call our readers attention to the advertisements of Messrs. Thomas Pullum & Co., Hirt & Brown, J. L. & A. J. Pinson, and E. M. Berry, now Collier & Berry. These houses are all reliable, and the drugs they dispense are pure.

*H. Planten & Son* advertise their well-known Capsules, both filled and empty. We can endorse these preparations from our personal experience.

*Howard's Extract of Gelseminum* is one of the most reliable preparations in the market. See Dr. Howard's advertisement.

*Pneumatic Aspiration* is coming into general use so rapidly that no physician can afford to do without an instrument. Codman & Shurtleff make a very fine one, and their price list is reasonable. See their advertisement for further particulars.

We confine our notices strictly to the drug and instrument line; but our readers will find it much to their advantage to look over our general advertisements as well. The same rule of reliability applies to all of our advertisements. H. B. L.

*Payments.*

THOSE who have remitted their dues since the mailing of the July number will appear in the September number. P.

